# The Prevalence of Gingival Recession According to the Cairo Classification in a Population from the North of Portugal

**DOI:** 10.3390/dj12120376

**Published:** 2024-11-22

**Authors:** Beatriz Moura, Filomena Salazar, Rosana Costa, Cristina Cabral, Cátia Reis

**Affiliations:** 1 Department of Medicine and Oral Surgery, University Institute of Health Sciences (IUCS-CESPU), 4585-116 Gandra, Portugal; a28195@alunos.cespu.pt (B.M.); filomena.salazar@iucs.cespu.pt (F.S.); rosana.costa@iucs.cespu.pt (R.C.); catia.reis@iucs.cespu.pt (C.R.); 2 Oral Pathology and Rehabilitation Research Unit (UNIPRO), University Institute of Health Sciences (IUCS-CESPU), 4585-116 Gandra, Portugal

**Keywords:** gingival recession, risk factors, prevalence, aesthetics, periodontal diseases and conditions

## Abstract

**Background/Objectives:** Gingival recession (GR) has a multifactorial etiology, resulting from the interaction of various factors. It affects aesthetics and comfort, and has negative consequences for quality of life. The main objective of this study is to investigate the prevalence of gingival recession in a sample of the population in northern Portugal, according to the classification system proposed by Cairo. The secondary objective is to investigate possible risk factors for the prevalence of gingival recession. **Methods:** This observational study analyzed 50 patients who met all our inclusion criteria. Participants underwent a clinical periodontal examination and completed a questionnaire. **Results:** On the lingual/palatine side, recession type 2 (RT2) was the most prevalent (39.1%), and on the buccal side, recession type 3 (RT3) was the most prevalent (37.7%). Statistically significant differences were found in the occurrence of RT3 on both sides, buccal and lingual/palatine, which was higher in patients over 65. Buccal side RT2 and buccal and lingual/palatine side RT3 were more prevalent in males. Buccal side RT2 was more prevalent in ex-smokers compared to nonsmokers. RT3 was more prevalent in ex-smokers compared to smokers and nonsmokers. Most patients have gingival recession with loss of interproximal attachment. **Conclusions:** Older age, male gender, high plaque levels, and smoking habits are considered risk factors.

## 1. Introduction

Gingival recession (GR) is defined as the apical migration of the gingival margin in relation to the cementoenamel junction (CEJ) and is associated with clinical attachment loss (CAL) and exposure of the root surface in the oral cavity [1].

Epidemiological studies have reported GR prevalence rates ranging from 40% to 100%, depending on the population and assessment methods used, indicating that GR is a common clinical finding [2]. GR has a multifactorial etiology, resulting from the combined action of several factors, including traumatic tooth brushing, inadequate orthodontic treatment, poor dental position, defective restorations affecting the gingival margin, gingival inflammation induced by bacterial plaque, a thin gingival phenotype, and the presence of periodontitis [1,3,4,5,6,7]. Epidemiological studies have identified risk factors such as gender, smoking, and age [1,8]. GR prevalence, extent, and severity increase with age, and males are more affected than females [3,8,9]. The presence of GR in the younger population is generally localized, and a generalized distribution is observed in older individuals, suggesting a cumulative effect and association with several factors, including a history of periodontal disease (PD) [3]. The frequency in adults with at least one GR ≥ 1 mm was 58% in the United States, 60.3% in Greece, 69.7% in Colombia, 84.9% in France, and 89.7% in Pomerania, a province in Eastern Germany [6]. Brazil achieved the highest percentage, with 99.7% [6].

A 2016 cross-sectional study in Portugal found an 85.1% prevalence of GR. It was observed that as the plaque index (PI) increased, with values between 75 and 100%, all patients presented GR [9]. Different gingival phenotype (GP) tends to react differently to inflammatory, traumatic, or surgical attacks [10]. Thick GP is generally associated with a state of ideal periodontal health, whereas thin GP is correlated with a higher incidence of periodontal complications [11]. Although there is no proven evidence, any differences in the risk of GR between thin and thick tissues may be more directly attributed to the triggering factors involved than to the GP itself, since in the absence of these factors the gingival margin (GM) in the thin GP may remain unchanged [12]. In patients with thin gingival phenotype (GP), traumatic tooth brushing can cause gingival recession (GR), while insufficient cleaning can lead to localized inflammation resulting in GR [13]. GR is a public health issue with psychosocial impacts, affecting aesthetics, comfort, and quality of life [14,15]. It can cause dental hypersensitivity and root surface lesion [4,8,15,16].

At the last World Workshop for the classification of periodontal and peri-implant diseases and conditions held in 2017, the Cairo classification was adopted as the new GR classification system [17]. Cairo defined three types of GR based on the level of interproximal CAL as the main criteria for diagnosis and prediction of root coverage [1]. In recession type 1 (RT1), there is no interproximal CAL; in recession type 2 (RT2), the loss of interproximal CAL is equal to or less than the loss of buccal CAL; finally, in recession type 3 (RT3), the interproximal CAL is larger than the buccal CAL [15,16].

In Europe, there are no representative data on the prevalence and risk factors of GR according to the new World Workshop classification [6]. To date, only a limited amount of data is available for a comprehensive assessment of the periodontal status of the Portuguese population [18]. There is a great need to carry out additional studies on GR in the country, given the influence it has on oral health [9].

This study’s main objective is to investigate the prevalence of RT1, RT2, and RT3 in a population sample from the north of Portugal, according to the classification system proposed by Cairo [17] and based on established guidelines by the last World Workshop for the classification of periodontal and peri-implant diseases and conditions [19]. The secondary objective is to investigate possible risk factors for the prevalence of GR.

## 2. Materials and Methods

### 2.1. Type of Study

This observational study is reported according to STROBE guidelines [20]. This study analyzed patients living in the north of Portugal, who attended dental appointments at CESPU-University Clinic in 2024. The interventions were approved by the ethics committee of the University of Health Sciences (reference: 23/CE-IUCS/2024) and conducted in accordance with the provisions of the Declaration of Helsinki.

### 2.2. Patient Selection

This study involved the participation of 50 patients who attended dental appointments at CESPU-University Clinic between June and September 2024. Participants were carefully informed through oral and written explanations about the objectives of the study and the associated risks and benefits. Informed consent was obtained from all participants included in the study. Patients were recruited for age, gender, and smoking habits. Ex-smokers were those who had quit smoking five years ago [21].

For inclusion in this study, patients had to be at least 18 years of age, of either gender, with single or multiple gingival recessions in the oral cavity. Excluded from this study were patients who received scaling and root planning or periodontal treatment during the last six months; subjects who did not present a complete periodontal clinical record and questionnaire, as well as free and informed consent to participate in the investigation; systemically compromised patients; patients currently undergoing orthodontic treatment or who had undergone it in the past; subjects under medication that can change the gingival contour, such as cyclosporine, phenytoin, calcium channel antagonists, and corticosteroid therapy; patients with human immunodeficiency virus (HIV); radiotherapy cancer patients; and pregnant women.

### 2.3. Periodontal Assessment

Before the periodontal examination, a questionnaire was carried out to obtain information on personal data (age and gender), family history, dental history, the patient’s oral health status, usual medication, and smoking habits. Measurements of the periodontal parameters were repeated in 30% of the sample, and intra- and interexaminer reliability was demonstrated using two independent examiners (B.M. and C.R.). A periodontal chart was carried out in order to collect the following variables: periodontal probing depth (PPD); the depth from the free gingival margin to the base of the sulcus, measured in millimeters at several locations around the whole circumference of the tooth; recession (REC): determined in millimeters as the distance from CEJ to the GM; clinical attachment loss (CAL), calculated as (CAL = PD + REC) average loss of total adhesion [22]; bleeding index (BOP; Ainamo and Bay [23]): presence or absence of bleeding on probing; plaque index (PI; O’Leary [24]): presence or absence of visible bacterial plaque, determined with the aid of a clinical probe or staining with plaque revealing. All periodontal examinations were performed with a manual probe (CP12) using gentle pressure. They were recorded in six locations per tooth (mesiobuccal, buccal, distobuccal, mesiolingual, lingual, and distolingual). In this study, third molars were also included. The height of the keratinized gingival tissue (KGT) was also measured, obtained by the distance between the GM and the mucogingival junction (MGJ) with the same periodontal probe. The GP was measured according to the transparency on probing. The periodontal probe was introduced into the buccal groove of the upper central incisors, upper laterals, or upper canines. Subsequently, the gingival phenotype was classified as thin if it was possible to visualize the periodontal probe through the sulcus and as thick if it was not possible. The present gingival recessions were classified according to the Cairo proposal and adopted by the new classification [19]: RT1 without loss of interproximal attachment; RT2 with loss of interproximal attachment, less than or equal to the loss of buccal attachment; RT3 when the loss of interproximal adhesion exceeds the loss of buccal adhesion [15].

### 2.4. Statistical Analysis

Statistical analysis involved descriptive statistics measures (absolute and relative frequencies, means, and respective standard deviations) and inferential statistics. In this, the Pearson correlation coefficient, the Fisher test, the Mann–Whitney U-test (MU), and the Kruskal–Wallis test were used. Distribution normality was analyzed using the Shapiro–Wilk test. The significance level to reject the null hypothesis was set at α ≤ 0.05. Statistical analysis was performed using SPSS^®^ (Statistical Package for the Social Sciences) software, version 28 for Windows.

## 3. Results

### 3.1. Characterization of the Sample According to Sociodemographic and Clinical Variables 

Table 1 shows the sample characterization. The sample consisted of data from 50 patients. The average age was 58.7 years, varying between a minimum of 25 and a maximum of 86 years. The majority were in the age group > 65 years (40%). Of the patients included in this study, 25 were male (50%) and 25 were female (50%). Regarding smoking habits, the majority were nonsmokers (56.0%), followed by ex-smokers (24.0%), and smokers (20.0%).

Table 2 shows the most prevalent gingival recessions in the sample according to the Cairo classification. On the lingual/palatine (L) side, RT2 is the most prevalent (39.1%), followed by RT3 (37.7%) and RT1 (23.2%). On the buccal (B) side, RT3 is the most prevalent (37.7%), followed by RT2 (37.2%), and the least prevalent is RT1 (25.1%).

Table 3 exhibits the gingival recessions, the height of keratinized gingiva, and the plaque index in the sample. RT3-B had the highest average occurrence of 2.74 ± 3.12 (0–15), followed by RT2-B with an average of 2.70 ± 2.75 (0–14). RT1-L had the lowest mean in the sample of 1.32 ± 1.94 (0–7). The keratinized tissue width (KTW) presented an average in millimeters (mm) of 5.40 ± 1.262 (2–10). The PI (%) presented an average of 55.05 ± 34.45 (0–100%).

Table 4 shows the patients’ gingival phenotype. The thick GP was the most prevalent in the sample, with 56.0% (N = 28), and the thin GP had a prevalence of 44.0% (N = 22), according to Table 4.

### 3.2. Relationship Between Gingival Recession and Different Risk Factors

#### 3.2.1. Relationship Between Gingival Recessions and Age

Table 5 shows the relationship of gingival recession according to different age groups. We found the following statistically significant differences:

RT3-L, with a significantly higher value in patients > 65 years old when compared to younger patients (2.75 vs. 1.69), X 2 KW (2) = 7.291, *p* = 0.026.
RT3-B, with a significantly higher value in patients > 65 years old when compared to younger patients (3.50 vs. 2.19), X 2 KW (2) = 7.800, *p* = 0.020.

#### 3.2.2. Relationship Between Gingival Recessions and Gender

Table 6 shows the relationship between gingival recession and gender. We found the following statistically significant differences:

RT2-B, with a significantly higher value in male patients (2.37 vs. 2.12), MU = 197.000, *p* = 0.023.
RT3-L, where the value is significantly higher in male patients (3.04 vs. 1.24), MU = 182.000, *p* = 0.009.
RT3-B, where the value is significantly higher in male patients (3.44 vs. 2.04), MU = 182.000, *p* = 0.036.

#### 3.2.3. Relationship Between Gingival Phenotype and Gender

Table 7 shows the relationship of gingival phenotype and gender. The thick GP is more prevalent in males (68.0%) when compared to females (44.0%). In relation to the thin GP, there is a higher prevalence in females (56.0%) when compared to males (32.0%), although the difference is not statistically significant (Fisher’s test, *p* = 0.154).

#### 3.2.4. Relationship Between Gingival Recessions and Gingival Phenotype

Table 8 shows the relationship of gingival recession and gingival phenotype. We found the following statistically significant differences:

RT3-L, the value is significantly higher in patients with thick GP (2.71 vs. 1.41), MU = 181.000, p = 0.010.
RT3-B, the value is significantly higher in patients with thick GP (3.21 vs. 2.14), MU = 196.500, p = 0.027.

#### 3.2.5. Relationship Between the Height of the Keratinized Gingiva and the Gingival Phenotype

Table 9 shows the relationship between the height of keratinized tissue and gingival phenotype. The width of keratinized tissue is higher in patients with thick gingival phenotype (5.46 vs. 5.32), although the difference is not statistically significant, MU = 247.500, *p* = 0.205.

#### 3.2.6. Plaque Index and Age

Table 10 shows the relationship between plaque index according to different age groups. The plaque index is higher in patients over 56–66 years old, although the differences are not statistically significant; X 2 KW (2) = 2.157, *p* = 0.340.

#### 3.2.7. Plaque Index and Gender 

Table 11 shows the relationship between plaque index and gender. The plaque index is higher in male patients, although the difference is not statistically significant; MU = 271.500, *p* = 0.425.

#### 3.2.8. Gingival Recession and Smoking Habits

We found the following statistically significant differences:

RT2-B, the value is significantly higher in ex-smokers when compared to nonsmokers (3.67 vs. 2.29), X 2 KW (2) = 6430,* p* = 0.040.
RT3-B, the value is significantly higher in ex-smokers when compared to nonsmokers and smokers (4.50 vs. 2.57 and 1.10), X 2 KW (2) = 10,738, *p* = 0.040.

Table 12 shows the relationship between gingival recession and smoking habits.

#### 3.2.9. Patient Perception of Gingival Recession

Of the 50 patients who had at least one GR, the majority, 64% (N = 32), stated that they did not have any type of perception of the presence of GR in the oral cavity and were unaware of its meaning. Only a minority of 36% (N = 18) demonstrated knowledge and awareness of the presence of GR in the oral cavity. According to this last group, 83.3% (N = 15) presented aesthetic concerns with the presence of GR in the oral cavity, and a minority of 16.7% (N = 3) reported not having any type of aesthetic concerns with its presence.

## 4. Discussion

Currently in Europe, there is a notable lack of representative data on the prevalence and risk factors of GR, according to the parameters established by the 2018 World Classification of Workshops [6]. At the same time, to date, a restricted amount of information has been made available for a comprehensive analysis of the periodontal status of the Portuguese population [18]. The present study is the first carried out in Portugal that evaluates the prevalence of gingival recessions according to the classification system proposed by Cairo [17] and its relationship with possible risk factors, aiming to expand the set of data available in Portugal.

The present study is based on the analysis of 647 buccal and lingual/palatine gingival recessions. It is possible to verify, by lingual/palatine, that RT2 (39.1%) is the most prevalent, followed by RT3 (37.7%) and the less-prevalent RT1 (23.2%). On the buccal side, RT3 and RT2 were the most prevalent (37.7% and 37.2%, respectively) and RT1 is the least prevalent (25.1%). These data are in line with a study carried out in the United States, which presented lower estimates of RT1 (12.4%), but a higher frequency of RT2 and RT3 (88.8% and 55.0%, respectively), among adults aged 30 and over [15]. In a study carried out in an urban population in northwestern Italy, RT1 had the most prevalent value (40.90%), followed by RT3 (36.68%) and RT2 (25.82%), respectively. The authors highlighted that it was the second study to use the classification system introduced by Cairo [6].

The thick GP was the most prevalent (56.0%) in the present study, and the thin GP had a prevalence of 44.0%. These data are in accordance with a study carried out in India, in which the thick GP was the most prevalent (56.75%) and the thin GP was the least prevalent in the sample (43.25%) [25]. In a study conducted in Saudi Arabia, a higher prevalence of the thick GP in the population (53%) and a lower prevalence of individuals with a thin GP (47%) were reported [26].

Data in the literature reported that the prevalence of GR increases with age and that it is more prevalent in males. Other epidemiological studies have shown that age is an important risk factor for GR [1]. The prevalence of GR varies among different populations and age groups. The studies by Susin et al. and Toker and Ozdemir demonstrated a prevalence of 50% to 99.7%, increasing with age in the Brazilian and Turkish populations [27,28]. These results are in accordance with our results showing that GR is increased with age. In an epidemiological study conducted in India, it was observed that the frequency of GR increases with age. It was possible to observe the lowest frequency of GR (26.9%) in the youngest age group (15–25 years) and the highest (70.27%) in the oldest age group (45–60 years) [3]. In the present study, the average occurrence of RT3 per buccal test is significantly higher in patients over 65 years of age compared to patients under 56 years of age (3.50 vs. 2.19). In RT3 per lingual/palatine, we can find a statistically significant higher value in patients over 65 years of age when compared to patients under 56 years of age (2.75 vs. 1.69). As evidenced in numerous epidemiological studies, the prevalence, extent, and severity of gingival recession tend to increase with advancing age, probably due to greater exposure to the etiological agent and predisposing factors. Furthermore, intrinsic age-related changes also play a synergistic role in the severity and extent of the condition [29]. The occurrence of GR in young patients is generally localized and appears to be more associated with isolated etiological factors. On the other hand, a more widespread distribution, such as that observed among older individuals, may indicate the associative and cumulative effect of several factors, including the presence of periodontal disease [9]. In the study mentioned above, a high frequency of GR was also discovered in men (60.5%) compared to women (39.5%), with statistical significance [3]. However, in a study carried out in Greece, the prevalence was slightly higher in men (51.8%) than in women (48.3%), but this difference was not statistically significant; in addition, a study carried out in a city in Iraq showed that, statistically, gingival recession among males was also not significantly greater than among females (46% versus 34.5%, respectively) [5,29]. Likewise, a statistically significant association between GR and gender was not detected in another study carried out in Iraq, in which the plaque index, the presence of periodontal diseases, and age were considered risk factors strongly related to GR [30]. In the present study, we discovered that RT2-B, RT3-B, and RT3-L have a significantly higher value in male patients when compared to female patients, which can be seen in Table 6.

Additionally, the thick gingival phenotype was more prevalent in males (68.0%) when compared to females (44.0%). In relation to the thin gingival phenotype, there is a higher prevalence in females (56.0%) when compared to males (32.0%), although the difference is not statistically significant. The results of the study are in line with another study carried out in India, which concluded that the thick phenotype was the most prevalent in the population and that there is no significant relationship with gender [25]. Divergently, a study carried out in the same country found statistically significant differences, with a greater prevalence of the thick phenotype in males and a greater prevalence of the thin phenotype in females [31].

The assessment of gingival phenotype is a common practice in clinical routine, both for epidemiological and therapeutic purposes. The thick gingival phenotype demonstrates resistance to trauma and is often associated with good periodontal health. For this, the predominant periodontal result is pocket formation, while for the thin gingival phenotype, it is more common to observe fenestration and dehiscence [26]. According to a study carried out in Karnataka, India, we can expect GR to be associated with a thinner phenotype; however, the study’s observations failed to report any relationship between gum thickness and the presence of GR [25]. In another study carried out in the same country, a higher prevalence of the thin phenotype (73.8%) was found in places with the presence of GR, although the results are not statistically significant [32]. The same happens in another investigation, in which no statistically significant association was found between gingival phenotype and GR in both groups of smokers and nonsmokers [33]. Contrary to a study carried out in Brazil, it was discovered that the smaller the thickness of the gum, the greater the degree of GR; however, due to the dispersion of the data, a low degree of Pearson’s correlation coefficient was observed between the variables [34]. In a study carried out in Greece, no statistically significant correlation between GP and GR was found, as the prevalence of GR was equally distributed between the two groups [5]. In the present study, statistically significant differences were found between RT3, both buccal and lingual/palatine, with a significantly higher value in patients with a thick GP compared to those with a thin GP. When older age is considered, GR due to PD becomes a more frequent phenomenon [5]. In the present study, the high presence of RT3 in the thick phenotype may be more directly related to the presence of periodontal disease than to the gingival phenotype itself. It has been proposed that the thick GP may be associated with a wider band of KGT [35]. It has been suggested that a wide zone of keratinized and attached gingiva is preferable to a narrow region or the total absence thereof, since a wider band would better withstand factors such as gingival inflammation, trauma caused by chewing, and tooth brushing [36]. In the present study, the height of the KGT is higher in patients with thick GP, although the difference is not statistically significant. One study reported a significant positive correlation observed between KGT height and GP, which supports the notion that patients with thin GP require more careful treatment planning [25]. In a study carried out in Nepal, the height of the KGT obtained significantly different values between the thin GP and the thick GP, with a greater amount of keratinized band present in the thick phenotype [10].

It is important to recognize that GR has multifactorial causes, and for this reason it is not ideal to consider just one factor as an isolated cause of this condition. Instead, it is necessary to recognize the interaction of several etiological antecedents that may simultaneously contribute to the occurrence of GR [29]. The presence of bacterial plaque is an important factor in the occurrence of GR; a study carried out in Iraq found a significant association between the presence of bacterial plaque and calculus with GR, and considered that, in addition to age, this would be one of the most important factors [30]. In the present study, the average PI in the sample was 55.05%, which we can consider high, as it represents more than half of the tooth surfaces with bacterial plaque. The PI was higher in male patients compared to female patients and in patients aged between 56 and 66 years, although the differences were not statistically significant.

Smoking is considered a risk factor associated with GR [1]. A study carried out in Greece demonstrated that the occurrence of GR is associated with smoking habits [37]. However, another study carried out in the same country was unable to establish an association between smoking and the presence of GR [5]. A study carried out in the United States population using the Cairo classification found that the prevalence of RT1 decreased in smokers and was justified by the increasing increase in periodontitis in the population [15]. In the present study, we found that the mean RT2-B and RT3-B are significantly higher in ex-smoker patients when compared to those who have never smoked. RT3-V has a higher value in ex-smokers when compared to current smokers. The difference may be related to the amount and duration of smoking that ex-smoking patients had when compared to smoking patients in the sample, which may be due to smoking for fewer years or in smaller quantities. The study does not invalidate the fact that there is a statistically significant greater relationship in current smokers when compared to ex-smokers. Therefore, in this study, we consider that the duration of the smoking habit and the daily quantity may have a significant impact on the occurrence of GR, and for this reason, more studies should be carried out with a larger sample in this regard.

According to a study carried out in 2019, and to the authors’ knowledge, only one other previous study investigated patients’ perception of GR [38]. According to the research carried out in this study, we can agree that there is a lack of data in this area. In a study carried out in private practice, 92% of gingival recessions were asymptomatic and/or not noticed by patients, and only 11% of patients requested treatment [14]. In another study, of the 98 patients who had at least one GR, 36% did not notice the presence of GR in the oral cavity and 64% were aware of the presence of GR. Of those who were aware, 49% were not concerned about GR, and only 24% were concerned about aesthetics [38]. In the present study, we can conclude that of the 50 patients who had at least 1 GR, 64% reported having no perception of the GR and were unaware of its meaning. Only a minority of 36% demonstrated that they were aware of the presence of GR in the oral cavity. In this last group, 83.3% had aesthetic concerns, and a minority of 16.7% did not have this concern.

We can attribute several positive points to this study with the implementation of a rigorous methodology, using the Cairo classification according to the World Workshop for the Classification of Periodontal and Peri-Implant Diseases and Conditions of 2017, enabling future comparisons with other studies at the same level worldwide. As this is a recent classification and there are a limited number of studies available, this study aims to expand the available data.

The limitations of the study were the limited sample size, the limited concentration of ages in the sample, and the method chosen to assess the gingival phenotype, which may be debatable, although it is a technique applied by several clinical studies that have investigated this parameter and confirmed the accuracy and repeatability of this method in daily clinical practice.

## 5. Conclusions

Most patients present GR with interproximal attachment loss, and RT3-B and RT2-L were the most prevalent type of recessions in this study. Older age, male gender, increased PI, and smoking habits are considered risk factors. Patients reported not having any perception of GR in the oral cavity and being unaware of its meaning. There is a need at the community level to create oral health awareness campaigns focusing on oral hygiene, plaque reduction, and smoking habits. Gingival recession has a complex etiology, which means that multiple factors together always contribute to its progression. More studies involving this classification are needed in order to compare them with other studies as reliably as possible.

## Figures and Tables

**Table 1:** Sample characterization (N = 50).

	M ^2^	SD ^3^
**Age**	58.7	15.2
	**N ^1^**	**%**
**Age groups**		
<56	16	32.0
56–65	14	28.0
>65	20	40.0
**Gender**		
Feminine	25	50.0
Masculine	25	50.0
**Smoking habits**		
Nonsmokers	28	56.0
Ex-smokers	12	24.0
Smokers	10	20.0

^1^ Sample number; ^2^ mean; ^3^ standard deviation.

**Table 2:** Gingival recessions characterization according to their type and tooth face.

	N	%
RT1-L ^1^	66	23.2
RT2-L ^2^	111	39.1
RT3-L ^3^	107	37.7
Total	284	100.0
	**N**	**%**
RT1-B ^4^	91	25.1
RT2-B ^5^	135	37.2
RT3-B ^6^	137	37.7
Total	363	100.0

N: sample number; ^1^ lingual/palatine type 1 recession; ^2^ lingual/palatine type 2 recession; ^3^ lingual/palatine type 3 recession; ^4^ buccal type 1 recession; ^5^ buccal type 2 recession; ^6^ buccal type 3 recession.

**Table 3:** Gingival recessions, keratinized gingiva, and plaque index.

	Minimum	Maximum	Average	Standard Deviation
RT1-L ^1^	0	7	1.32	1.94
RT1-B ^2^	0	12	1.82	2.73
RT2-L ^3^	0	9	2.22	2.55
RT2-B ^4^	0	14	2.70	2.75
RT3-L ^5^	0	14	2.14	3.17
RT3-B ^6^	0	15	2.74	3.12
KTW ^7^	2	10	5.40	1262
PI ^8^ (%)	0	100.00	55.05	34.45

^1^ Lingual/palatine type 1 recession; ^2^ lingual/palatine type 2 recession; ^3^ lingual/palatine type 3 recession; ^4^ buccal type 1 recession; ^5^ buccal type 2 recession; ^6^ buccal type 3 recession; ^7^ keratinized tissue width; ^8^ plaque index.

**Table 4:** Gingival phenotype.

	N ^1^	%
Thick	28	56.0
Thin	22	44.0
Total	50	100.0

^1^ Sample number.

**Table 5:** Gingival recession according to different age groups.

	<56 Years Old		56–65 Years Old		>65 Years Old		
	M	SD	M	SD	M	SD	Sig.
RT1-L ^1^	1.19	1.80	1.21	2.33	1.50	1.85	0.517
RT1-B ^2^	2.44	2.50	1.36	2.56	1.65	3.05	0.171
RT2-L ^3^	1.81	2.34	1.29	1.64	3.20	2.98	0.098
RT2-B ^4^	2.25	3.71	2.50	1.79	3.20	2.46	0.119
RT3-L ^5^	1.69	3.84	1.79	2.83	2.75	2.86	0.026 *
RT3-B ^6^	2.19	3.97	2.29	2.92	3.50	2.42	0.020 *

M: mean; SD: standard deviation; * *p* < 0.05; ^1^ Lingual/palatine type 1 recession; ^2^ buccal type 1 recession; ^3^ lingual/palatine type 2 recession; ^4^ buccal type 2 recession; ^5^ lingual/palatine type 3 recession; ^6^ RT3-B: buccal type 3 recession.

**Table 6:** Gingival recessions and gender.

	Feminine		Masculine		
	M	SD	M	SD	Sig.
RT1-L ^1^	1.04	1.86	1.60	2.02	0.293
RT1-B ^2^	1.52	2.45	2.12	3.00	0.403
RT2-L ^3^	1.68	2.21	2.76	2.80	0.162
RT2-B ^4^	2.12	3.03	3.28	2.37	0.023 *
RT3-L ^5^	1.24	2.77	3.04	3.35	0.009 **
RT3-B ^6^	2.04	3.17	3.44	2.97	0.036 *

M: mean; SD: standard deviation; * *p* < 0.05 ** *p* < 0.01; ^1^ lingual/palatine type 1 recession; ^2^ buccal type 1 recession; ^3^ lingual/palatine type 2 recession; ^4^ buccal type 2 recession; ^5^ lingual/palatine type 3 recession; ^6^ buccal type 3 recession.

**Table 7:** Gingival phenotype and gender.

Gingival Phenotype		Gender		Total
		Feminine	Masculine	
Thick	Freq.	11	17	28
	% Gender	44.0%	68.0%	56.0%
Thin	Freq.	14	8	22
	% Gender	56.0%	32.0%	44.0%
Total	Freq.	25	25	50
	% Gender	100.0%	100.0%	100.0%

**Table 8:** Gingival recessions and gingival phenotype.

	Thick		Thin		
	M	SD	M	SD	Sig.
RT1-L ^1^	1.14	1.69	1.55	2.24	0.777
RT1-B ^2^	1.50	2.65	2.23	2.84	0.267
RT2-L ^3^	2.61	2.70	1.73	2.33	0.205
RT2-B ^4^	3.00	2.55	2.32	3.01	0.174
RT3-L ^5^	2.71	3.15	1.41	3.13	0.010 *
RT3-B ^6^	3.21	2.78	2.14	3.48	0.027 *

M: mean; SD: standard deviation; * *p* < 0.05; ^1^ lingual/palatine type 1 recession; ^2^ buccal type 1 recession; ^3^ lingual/palatine type 2 recession; ^4^ buccal type 2 recession; ^5^ lingual/palatine type 3 recession; ^6^ buccal type 3 recession.

**Table 9:** Height of keratinized gingival tissue and gingival phenotype.

	Thick		Thin		
	M	SD	M	SD	Sig.
KTW	5.46	1.43	5.32	1.04	0.205

M: mean; SD: standard deviation; KTW: keratinized tissue width.

**Table 10:** Plaque index and age.

	<56 Years Old		56–65 Years Old		>65 Years Old		
	M	SD	M	SD	M	SD	Sig.
PI (%)	54.16	41.09	66.29	32.14	47.91	29.56	0.340

M: mean; SD: standard deviation; PI: plaque index.

**Table 11:** Plaque index and gender.

	Feminine		Masculine		
	M	SD	M	SD	Sig.
PI (%)	51.16	35.73	58.94	33.39	0.425

M: mean; SD: standard deviation; PI: plaque index.

**Table 12:** Gingival recession and smoking habits.

	Nonsmokers		Ex-Smokers		Smoking		
	M	SD	M	SD	M	SD	Sig.
RT1-L ^1^	1.32	1.96	1.33	1.97	1.30	2.06	0.997
RT1-B ^2^	1.36	2.20	3.25	3.91	1.40	1.96	0.300
RT2-L ^3^	2.00	2.68	2.08	2.19	3.00	2.71	0.338
RT2-B ^4^	2.29	3.24	3.67	2.06	2.70	1.70	0.040 *
RT3-L ^5^	2.07	3.42	3.50	3.42	0.70	0.82	0.052
RT3-B ^6^	2.57	3.43	4.50	2.65	1.10	1.45	0.005 **

M: mean; SD: standard deviation; * *p* < 0.05 ** *p* < 0.01; ^1^ lingual/palatine type 1 recession; ^2^ buccal type 1 recession; ^3^ lingual/palatine type 2 recession; ^4^ buccal type 2 recession; ^5^ lingual/palatine type 3 recession; ^6^ buccal type 3 recession.

## Data Availability

The data can be accessed by contacting the corresponding author.
